# High-Dose Benzodiazepines Positively Modulate GABA_A_ Receptors via a Flumazenil-Insensitive Mechanism

**DOI:** 10.3390/ijms23010042

**Published:** 2021-12-21

**Authors:** Na Wang, Jingjing Lian, Yanqing Cao, Alai Muheyati, Shanshan Yuan, Yujie Ma, Shuzhuo Zhang, Gang Yu, Ruibin Su

**Affiliations:** State Key Laboratory of Toxicology and Medical Countermeasures, Beijing Key Laboratory of Neuropsychopharmacology, Beijing Institute of Pharmacology and Toxicology, Beijing 100850, China; 18369610393@163.com (N.W.); alianjingjing@163.com (J.L.); cyqorange@163.com (Y.C.); 18097543868@163.com (A.M.); dr_yuanshanshan@163.com (S.Y.); myj31014@163.com (Y.M.); Zhangshuzhuo9@hotmail.com (S.Z.)

**Keywords:** GABA_A_ receptors, benzodiazepine, non-classical binding site, voltage-clamp electrophysiology, loss of righting reflex

## Abstract

Benzodiazepines (BZDs) produce versatile pharmacological actions through positive modulation of GABA_A_ receptors (GABA_A_Rs). A previous study has demonstrated that high concentrations of diazepam potentiate GABA currents on the α_1_β_2_γ_2_ and α_1_β_2_ GABA_A_Rs in a flumazenil-insensitive manner. In this study, the high-concentration effects of BZDs and their sensitivity to flumazenil were determined on synaptic (α_1_β_2_γ_2_, α_2_β_2_γ_2_, α_5_β_2_γ_2_) and extra-synaptic (α_4_β_2_δ) GABA_A_Rs using the voltage-clamp electrophysiology technique. The in vivo evaluation of flumazenil-insensitive BZD effects was conducted in mice via the loss of righting reflex (LORR) test. Diazepam induced biphasic potentiation on the α_1_β_2_γ_2_, α_2_β_2_γ_2_ and α_5_β_2_γ_2_ GABA_A_Rs, but did not affect the α_4_β_2_δ receptor. In contrast to the nanomolar component of potentiation, the second potentiation elicited by micromolar diazepam was insensitive to flumazenil. Midazolam, clonazepam, and lorazepam at 200 µM exhibited similar flumazenil-insensitive effects on the α_1_β_2_γ_2_, α_2_β_2_γ_2_ and α_5_β_2_γ_2_ receptors, whereas the potentiation induced by 200 µM zolpidem or triazolam was abolished by flumazenil. Both the GABA_A_R antagonist pentylenetetrazol and Fa173, a proposed transmembrane site antagonist, abolished the potentiation induced by 200 µM diazepam. Consistent with the in vitro results, flumazenil antagonized the zolpidem-induced LORR, but not that induced by diazepam or midazolam. Pentylenetetrazol and Fa173 antagonized the diazepam-induced LORR. These findings support the existence of non-classical BZD binding sites on certain GABA_A_R subtypes and indicate that the flumazenil-insensitive effects depend on the chemical structures of BZD ligands.

## 1. Introduction

GABA_A_ receptors (GABA_A_Rs), the major inhibitory neurotransmitter receptors in the nervous system, are complex receptors that are critically involved in numerous physiological and pathological processes. They are heterogeneous pentamers assembled from at least 19 subunits (α_1–6_, β_1–3_, γ_1–3_, δ, ε, π, ρ_1–3_, and θ), and there are potentially dozens of active GABA_A_R subtypes with distinct distributional and functional characteristics [1,2,3,4]. Ternary receptors composed of two α, two β, and one γ (or δ) subunits are considered to constitute the majority of GABA_A_Rs. On the other hand, the complexity of GABA_A_Rs lies in various allosteric modulatory sites [5,6]. Although knowledge concerning the GABA_A_R structure and its interaction with various ligands is increasing rapidly [5,6,7,8,9,10,11], a complete elucidation of the modulatory mechanisms of GABA_A_R is still far from being achieved.

As one of the most important GABA_A_R allosteric modulators, benzodiazepines (BZDs) have wide and versatile clinical applications. At low or high dosages, BZDs, such as diazepam, produce anti-anxiety, anti-seizure, sedation, and anesthetic effects [12]. Conclusive evidence has shown that the anti-anxiety and sedative effects of BZDs are associated with the high-affinity (classical) sites located at the α+/γ− interface of synaptic GABA_A_Rs [13,14]. However, whether BZD-induced anesthesia is mediated by the classical binding sites remains to be clarified.

In addition to the classical binding sites, several new binding sites of BZDs on GABA_A_R are proposed [15,16,17,18]. In particular, a transmembrane BZD binding site that is activated by micromolar concentration of diazepam was suggested on recombinant α_1_β_2_γ_2_ and α_1_β_2_ GABA_A_Rs. However, these in vitro experiments were conducted using limited BZDs and α_1_-containing GABA_A_Rs. Previously, we demonstrated the flumazenil-insensitive BZD effects in a series of αβ binary GABA_A_Rs [19] and in zebrafish larvae [20]. By using the electrophysiological technique, the present study revealed flumazenil-insensitive diazepam modulation on a series of ternary GABA_A_Rs, which was abolished by both the GABA_A_R antagonist pentylenetetrazol and Fa173, a proposed GABA_A_R antagonist targeting the transmembrane site [21]. The effects of structurally differentiated BZDs were further compared to verify the objectivity and universal significance of the non-classical site. Both pentylenetetrazol and Fa173, but not flumazenil, antagonized BZD-induced anesthesia, which is considered to be related to high-dose BZD effects. The present study provides novel evidence supporting the existence of a flumazenil-insensitive mechanism in BZD modulation of GABA_A_Rs.

## 2. Results

### 2.1. Diazepam Exhibits Flumazenil-Insensitive Modulation of α_1_β_2_γ_2_, α_2_β_2_γ_2_, and α_5_β_2_γ_2_ GABA_A_Rs

**Figure 1:**
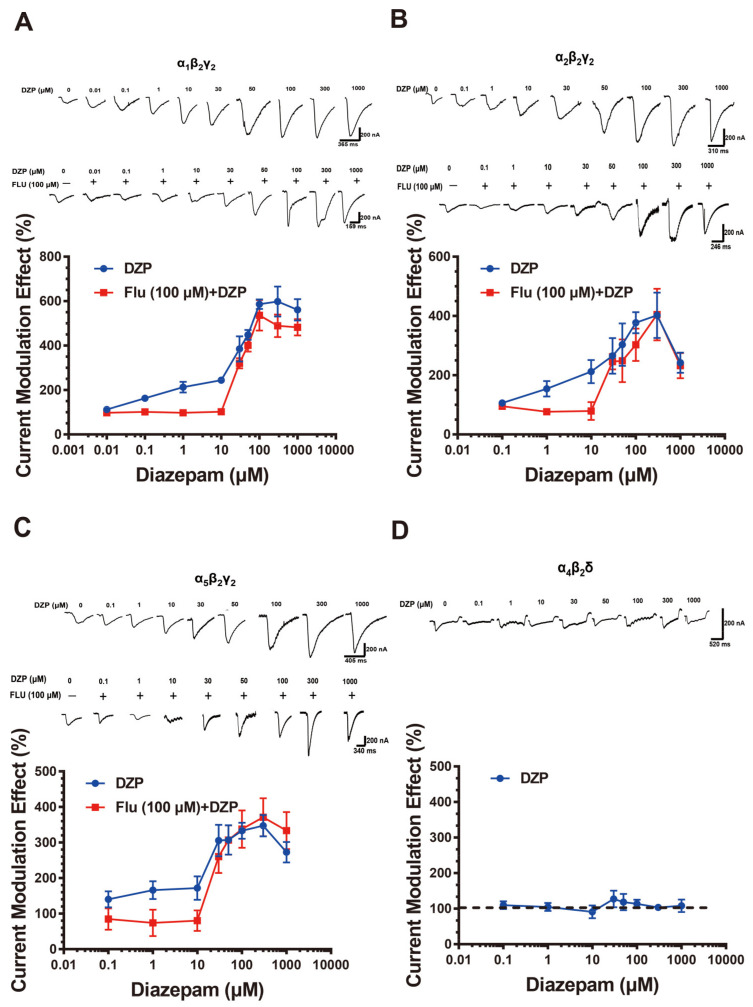
Diazepam modulated the α_1_β_2_γ_2_, α_2_β_2_γ_2_ and α_5_β_2_γ_2_ receptors in flumazenil-sensitive and flumazenil-insensitive manners. The concentration-response relationships of diazepam for modulating the α_1_β_2_γ_2_ (**A**), α_2_β_2_γ_2_ (**B**), α_5_β_2_γ_2_ (**C**), and α_4_β_2_δ (**D**) receptors were determined in the absence or presence of flumazenil. The GABA concentrations were 1, 0.1, 1, and 0.1 μM for α_1_β_2_γ_2_, α_2_β_2_γ_2_, α_5_β_2_γ_2_, and α_4_β_2_δ receptors, respectively. Representative current traces are shown on the top; data summary is shown on the bottom. Data represent mean ± SEM, *n* = 4–5.

The effects of diazepam on the α_1_β_2_γ_2_, α_2_β_2_γ_2_, α_5_β_2_γ_2_, and α_4_β_2_δ receptors were observed across a broad range of concentrations (0.1 to 1000 μM), in the presence of 1, 0.1, 1, and 0.1 μM GABA respectively (these concentrations elicited 3~10% of the maximal GABA currents, EC_3-10_). Consistent with the previous study [15], diazepam potentiated the GABA-elicited currents on the α_1_β_2_γ_2_ receptor in a biphasic manner (Figure 1A). The low- and high-concentration potentiation was at 0.1~10 and 10~1000 μM respectively, and the latter could not be antagonized by the classical binding site antagonist flumazenil (even at a concentration as high as 100 μM). Similarly, diazepam produced two components of potentiation in modulating the α_2_β_2_γ_2_ and α_5_β_2_γ_2_ receptors (Figure 1B,C). The first component of potentiation was induced by 0.1~10 μM diazepam with a maximum of ~200% at both receptors. At concentrations above 10 μM, diazepam evoked a second component of potentiation on the α_2_β_2_γ_2_ and α_5_β_2_γ_2_ receptors, further increasing GABA-elicited currents to approximately 400 and 350%, respectively. Flumazenil antagonized the action of diazepam at concentrations below 10 μM, but not above. However, diazepam did not significantly affect the α_4_β_2_δ receptor (Figure 1D).

### 2.2. Different BZD Ligands Exhibit Distinct Flumazenil-Insensitive Modulation of Different GABA_A_Rs

**Figure 2:**
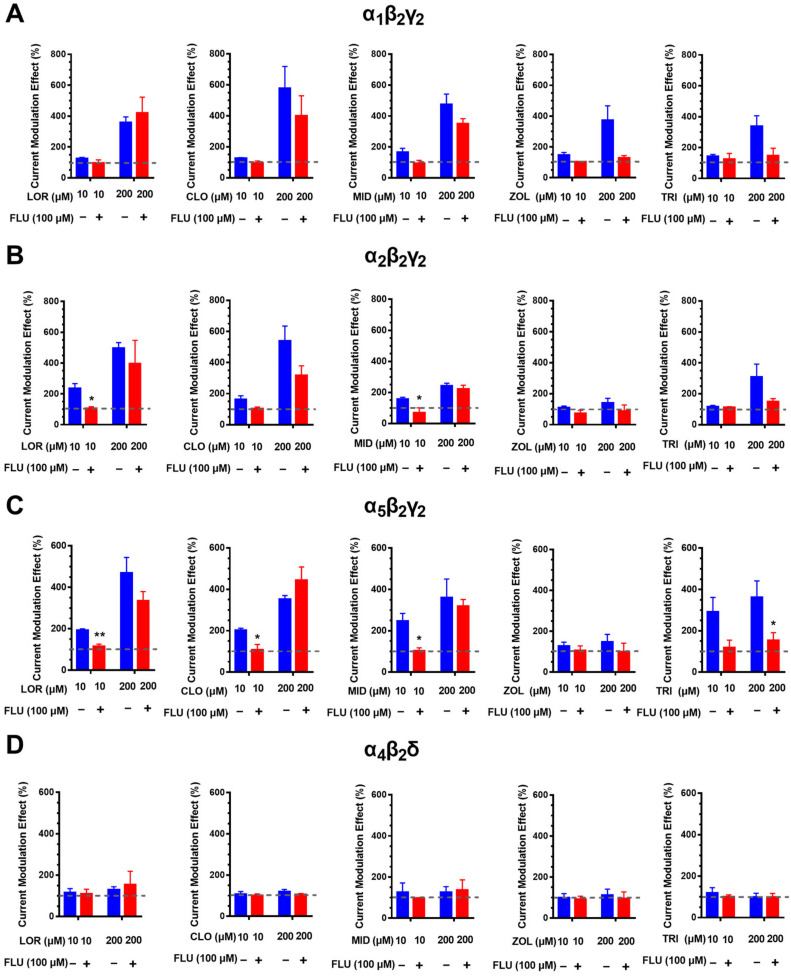
Different BZD ligands exhibited distinct modulation of ternary GABA_A_Rs. Effects of lorazepam (LOR), clonazepam (CLO), midazolam (MID), zolpidem (ZOL) and triazolam (TRI) in potentiating the GABA-elicited currents were evaluated at low (10 μM) and high (200 μM) concentrations in the absence or presence of flumazenil (100 μM) on the α_1_β_2_γ_2_ (**A**), α_2_β_2_γ_2_ (**B**), α_5_β_2_γ_2_ (**C**) and α_4_β_2_δ (**D**) receptors. The dotted lines indicate the basal levels without drug treatment. Data represent mean ± SEM, *n* = 4, * *p* < 0.05, ** *p* < 0.01, vs control group, via *t*-test.

Chemically differentiated BZD ligands, all of which bind to the classical α/γ sites, were tested to clarify whether the high-dose potentiation was exclusive to diazepam. The concentrations of 10 and 200 μM were selected to represent maxima of the low- and high-dose effects, respectively. Similar to diazepam, 10 μM of lorazepam, clonazepam (both belonging to the 1,4-BZDs) and midazolam (imidazo-benzodiazepine) potentiated the GABA-elicited currents on the α_1_β_2_γ_2_, α_2_β_2_γ_2_ and α_5_β_2_γ_2_ receptors, and these effects were abolished by flumazenil. The potentiation induced by 200 μM lorazepam, clonazepam or midazolam was even greater on these three GABA_A_R subtypes, and these effects were resistant to flumazenil (Figure 2). Zolpidem (imidazopyridine), which was shown to be selective for the α_1_β_2_γ_2_ receptor [22], produced a main and significant modulation on the α_1_β_2_γ_2_ receptor, but its effects at both 10 and 200 μM were antagonized by flumazenil. Triazolam (triazolo-benzodiazepine) positively modulated the α_1_β_2_γ_2_, α_2_β_2_γ_2_ and α_5_β_2_γ_2_ receptors, but its effects at both 10 and 200 μM were also substantially antagonized by flumazenil. In addition, all of the ligands tested exhibited limited modulatory effects on the α_4_β_2_δ receptor, at either 10 or 200 μM.

### 2.3. Pentylenetetrazol and Fa173 Abolish the Flumazenil-Insensitive Diazepam Effects

**Figure 3:**
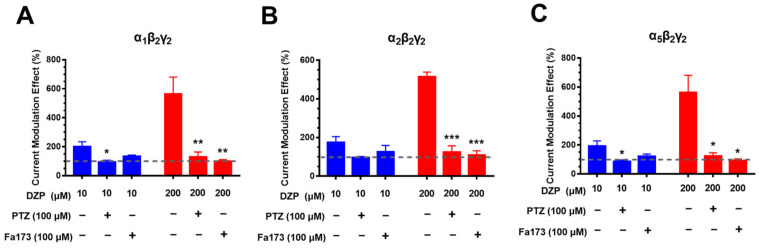
Pentylenetetrazol and Fa173 abolished the flumazenil-insensitive diazepam effects on ternary GABA_A_Rs. Effects of diazepam (DZP) in potentiating the GABA-elicited currents were evaluated at low (10 μM) and high (200 μM) concentrations in the absence or presence of pentylenetetrazol (PTZ, 100 μM) or Fa173 (100 μM) on the α_1_β_2_γ_2_ (**A**), α_2_β_2_γ_2_ (**B**) and α_5_β_2_γ_2_ (**C**) receptors. The dotted lines indicate the basal levels without drug treatment. Data represent mean ± SEM, *n* = 4, * *p* < 0.05, ** *p* < 0.01, *** *p* < 0.001 vs. control group, via *t*-test.

To exclude the possibility that the high-concentration BZD effects were a type of nonspecific effect, the effects of 200 μM diazepam were observed in the presence of either the GABA_A_R antagonist pentylenetetrazol or Fa173, a proposed transmembrane site antagonist [21]. Similarly, on the α_1_β_2_γ_2_, α_2_β_2_γ_2_ and α_5_β_2_γ_2_ receptors, diazepam produced two-graded potentiation on the GABA-elicited current at 10 and 200 μM. 100 μM PTZ or Fa173 antagonized not only the low-concentration but also the high-concentration effects of diazepam (Figure 3). These results indicated that the flumazenil-insensitive effects are specifically mediated by GABA_A_Rs, and possibly via the transmembrane binding sites.

### 2.4. Anesthesia Induced by Diazepam and Midazolam, but Not Zolpidem, Is Resistant to Flumazenil

**Figure 4:**
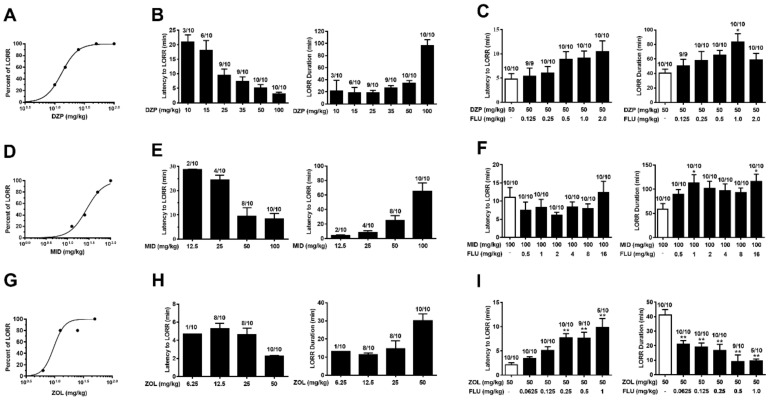
Flumazenil antagonized LORR induced by zolpidem, but not by diazepam or midazolam. A series of doses of diazepam (DZP), midazolam (MID) and zolpidem (ZOL) were administered intraperitoneally, and percentage of LORR (**A**,**D**,**G**) and latency to and duration of LORR (**B**,**E**,**H**) were recorded. Flumazenil (FLU) was intravenously injected immediately before BZD administration, and its effects on LORR induced by 50 mg kg^−1^ diazepam, 100 mg kg^−1^ midazolam or 50 mg kg^−1^ zolpidem were evaluated (**C**,**F**,**I**). Data represent mean ± SEM, *n* = 9−10. Figure on top of each bar is the number of mice that lost the righting reflex over the total number of the mice tested. One-way ANOVA; * *p* < 0.05, ** *p* < 0.01 *vs* control group, according to post hoc analysis with Dunnett’s multiple comparison test.

The high-dose effects of BZD ligands were evaluated in vivo by using LORR as an index of anesthesia. Diazepam, midazolam and zolpidem were chosen to compare the potential flumazenil-insensitive effects of different ligands. All three ligands dose-dependently induced LORR in mice (Figure 4A,D,G), and an increase in BZD dose led to a decrease in latency to and an increase in duration of LORR. Complete (100%) LORR was caused by diazepam, midazolam and zolpidem at doses of 50, 100 and 50 mg kg^−1^, respectively. Flumazenil treatment failed to antagonize LORR induced by diazepam or midazolam. Moreover, flumazenil even prolonged the duration of LORR at some doses (Figure 4C,F). In contrast, zolpidem-induced anesthesia was significantly antagonized by flumazenil. Flumazenil dose dependently increased the latency (F (5, 53) = 9.82, *p* < 0.01) and reduced the duration (F (5, 53) = 11.06, *p* < 0.01) of LORR induced by zolpidem. In addition, the percentage of zolpidem-induced LORR was reduced to 50% by flumazenil at a dose of 1 mg kg^−1^ (Figure 4I).

### 2.5. Diazepam-Induced Anesthesia Is Antagonized by Pentylenetetrazol and Fa173

**Figure 5:**
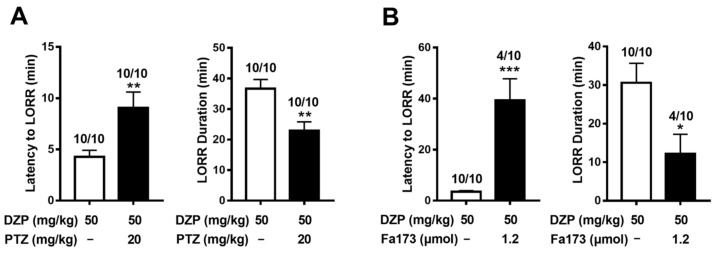
Pentylenetetrazol and Fa173 antagonized diazepam-induced LORR. Pentylenetetrazol (PTZ, 20 mg kg^−1^, i.v.) or Fa173 (1.2 μmol/mouse, i.c.v.) was injected immediately before diazepam (DZP, 50 mg kg^−1^, i.p.) administration. The effect of pentylenetetrazol (**A**) or Fa173 (**B**) on latency to and duration of diazepam-induced LORR were recorded. Data represent mean ± SEM, *n* = 10. Figure on top of each bar is the number of mice that lost the righting reflex over the total number of the mice tested. * *p* < 0.05, ** *p* < 0.01, *** *p* < 0.001 *vs* control group, via *t*-test.

Pentylenetetrazol and Fa173 were further used to determine the specific target for the high-dose BZD effects in living animals. Compared with the control, pentylenetetrazol treatment significantly increased the latency to (t = 2.88, *p* < 0.01, Figure 5A) and reduced the duration (t = 3.3, *p* < 0.01) of LORR induced by diazepam, indicating that the high-dose BZD effects were mediated via GABA_A_Rs. Similarly, Fa173 treatment antagonized diazepam-induced LORR, resulting in significant decreases in LORR percentage (*p* < 0.05, Figure 5B) and duration (t = 2.571, *p* < 0.05), and an increase in LORR latency (t = 4.247, *p* < 0.001). This was consistent with the in vitro results of Fa173 and suggested that high-dose diazepam activates the transmembrane binding sites of GABA_A_Rs.

## 3. Discussion

The present study demonstrated that high doses of BZDs produced profound and flumazenil-insensitive potentiation of GABA-elicited currents on certain GABA_A_R subtypes. Consistent with the electrophysiological observations, the anesthesia induced by diazepam, the BZD phenotype, was resistant to flumazenil, but antagonized by pentylenetetrazol or Fa173. The findings of this study support the existence of a non-classical mechanism in GABA_A_R modulation that may contribute to BZD-induced anesthesia. 

Direct experimental evidence is still lacking concerning BZD concentration around synaptic and extra-synaptic GABA_A_Rs after systemic administration, although it is important to determine the clinical significance of high-dose BZD effects. Based on the measured plasmic levels and their high lipid solubility, the concentrations of BZDs in the CNS were estimated to reach double-digit micromolar levels [23,24]. It is reasonable to speculate even higher levels of BZDs in specific brain regions and neural circuits under some circumstances of iatrogenic or self-inflicted overdose. Therefore, the local BZD concentrations were very likely to be high enough for flumazenil-insensitive potentiation under some practical conditions, especially in terms of BZD-induced anesthesia and intoxication.

In contrast to the extensive research on the high-affinity modulatory effects of BZD at nanomolar concentrations, very limited studies have addressed the effects and mechanisms of micromolar BZD. Moreover, there are controversial observations regarding the interaction of low- and high-affinity BZD effects. High-concentration flurazepam inhibited the GABA_A_R modulation mediated by the classical BZD binding site [15,25], while diazepam at concentrations above 20 µM further potentiated the α_1_β_2_γ_2_ receptor [15]. Our results are in good agreement with the latter, and extend the finding on diazepam and the α_1_β_2_γ_2_ receptor to a series of ligands and receptors. The flumazenil-insensitive high-dose effects of classical BZDs on synaptic GABA_A_Rs support the existence of non-classical binding sites, precluding the possibility that the micromolar potentiation of diazepam is a non-specific effect. The fact that Fa173 abolishes the high-dose BZD effects further verifies the non-classical binding sites, possibly located at the transmembrane domain (TMD) of GABA_A_Rs. However, other non-classical mechanisms may contribute to high-dose BZD effects [26].

The high-dose BZD effects may mean a broader and deeper depression of the central nervous system, which is considered to be related to general anesthesia [27]. In good agreement with previous studies [20], the full occupation of the non-classical binding sites by high concentrations of BZDs resulted in 2–3-fold increases in the maximum effects relative to modulation via the classical binding sites. Furthermore, the high-dose BZD effects are assumed to affect more GABA_A_R subtypes, as the construction of the non-classical binding sites does not require the γ subunit [19]. It is also interesting to see that the non-classical binding sites possessed similar dependence on the α subunit compared to the classical binding sites; i.e., α_1_-, α_2_- or α_5_-, but not α_4_-containing receptors were sensitive to BZD modulation. On the other hand, high-dose modulation was observed in classical BZDs but not non-BZD structures (such as zolpidem), although both categories of ligands bind to the classical binding sites of GABA_A_Rs. Similarly, a recent study has suggested that the structural features of BZD ligands govern their abilities to bind to the etomidate binding site of the GABA_A_Rs [28].

Flumazenil, which competes with BZDs to bind to the classical binding site, is the only specific therapeutic for BZD intoxication in clinic. Extensive evidence supports the effective antagonism of flumazenil on sedation, anti-anxiety, and anti-convulsion activity induced by relatively low doses of diazepam [29,30,31], while its effects on high-dose BZDs are rarely studied. The present study investigated the effectiveness of flumazenil against BZD-induced anesthesia, using the LORR as an in vivo model representing high-dose BZD effects. Flumazenil failed to effectively antagonize LORR induced by diazepam and midazolam. In particular, LORR duration was not reduced, but even prolonged by flumazenil under some doses. This result, which was consistent with the in vitro results, suggests that there is a flumazenil-insensitive mechanism in BZD-induced modulation of GABA_A_Rs, and that flumazenil may be inefficient in antagonizing some BZD effects, such as anesthesia, when used against high doses of certain BZDs.

The binding modes and mechanistic effects of BZD ligands were increasingly resolved, as the high-resolution structures of GABA_A_Rs in complex with BZDs were presented. Strong densities were observed in the TMD of the α_1_β_3_γ_2_ receptor, which is also considered the binding site for general anesthetics [9]. Recently, diazepam was demonstrated to share a binding site with anesthetics in the TMD of the α_1_β_2_γ_2_ receptor [11]. The binding of diazepam to this site may contribute to stabilization of the TMD and positively modulate the receptor in a similar way to anesthetics. These structural studies of GABA_A_Rs provided an excellent explanation for the flumazenil-insensitive BZD effects and the non-classical BZD modulatory mechanism.

In conclusion, the present study provided detailed evidence supporting the existence of flumazenil-insensitive BZD effects in a series of GABA_A_Rs and their potential association with BZD-induced anesthesia. These findings enhance the understanding of GABA_A_R modulation and BZD pharmacology, and suggest that some classical BZDs produce profound inhibition of brain function by binding to non-classical sites. Furthermore, elaborate analysis of the interaction of ligands with non-classical sites may prompt the development of novel drugs in modulating wakefulness and sleep [21,32].

## 4. Materials and Methods

### 4.1. Chemicals

GABA was obtained from Sigma-Aldrich (St. Louis, MO, USA). Flumazenil (PubChem ID: 3373), clonazepam (PubChem ID: 2802), lorazepam (PubChem ID: 3985), zolpidem (PubChem ID: 5732), triazolam (PubChem ID: 5556) were purchased from the National Institutes for Food and Drug Control (Beijing, China). Diazepam (PubChem ID: 3016), midazolam (PubChem ID: 4192), pentylenetetrazol (PubChem ID: 5917) were obtained from Jiangsu Enhua Pharmaceutical Co., Ltd. (Xuzhou, China). Fa173 was synthesized and provided by Beijing ShiKang Synthesis Pharmaceutical Co., Ltd. All drugs were dissolved in DMSO and stored at −20 °C and were diluted to the needed concentrations using recording solution at room temperature on the experimental day.

### 4.2. Expression of GABA_A_ Receptors in Xenopus Oocytes

The subcloning of human α_1_, α_2_, α_4_, α_5_, β_2_, γ_2_, and δ cDNAs into the pGH19 vector was performed as described previously [33]. The cRNA was transcribed from the linearized cDNAs via standard in vitro transcription procedures using the T7 mMESSAGE mMACHINE High Yield Capped RNA transcription kit (Invitrogen). Oocytes were harvested from anesthetized adult female *Xenopus laevis*, dispersed and incubated in oocyte Ringer (OR_2_, 82 mM NaCl, 2.5 mM KCl, 5 mM HEPES, 1 mM MgCl_2_, pH 7.6) plus 0.8 mg/mL collagenase A (Sigma, USA) for approximately 1 h. After isolation, the oocytes were thoroughly rinsed with OR_2_ and stage V or VI oocytes were separated and selected. On the next day, oocytes were injected with 40 ng of cRNA mixtures encoding α_1/2/5_β_2_γ_2_ and α_4_β_2_δ at a ratio of 1α:1β:1γ/δ. Injected oocytes were incubated at 18 °C in ND96 medium (96 mM NaCl, 2 mM KCl, 1.8 mM CaCl_2_, 1 mM MgCl_2_, 5 mM HEPES, pH 7.6). The oocytes expressing synaptic (α_1_β_2_γ_2_, α_2_β_2_γ_2_, α_5_β_2_γ_2_) and extra-synaptic (α_4_β_2_δ) GABA_A_Rs were used for electrophysiological recording 1–3 and 4–6 days after cRNA injection, respectively.

### 4.3. Two-Electrode Voltage Clamp Electrophysiology

Whole-cell currents were measured using the two-electrode voltage clamp technique. Microelectrodes were filled with 3 M KCl and those with resistance between 1.0–2.5 MΩ were used. Recordings were performed under constant perfusion at room temperature. Currents were amplified with an OC-725C (Warner Instruments, Hamden, USA) and digitized with a Digidata 1440 (Molecular Devices, San Jose, CA, USA) at 100 Hz. In all cases, currents in response to the application of drugs were recorded using Clampex 10.3 software (Axon Instruments, San Jose, CA, USA) and data were sampled at 2 kHz and filtered at 0.5 kHz. A gap-free protocol was applied with the holding membrane potential at −70 mV. Each drug application was followed by a washout in bath solution (approximately 5 min).

### 4.4. Mouse Behavioral Test

Adult male Kunming mice ages 3–4 weeks (19–21 g) were obtained from Beijing Animal Center (Beijing, China). Animals were housed 10 per cage with free access to food and water on a 12 h light–dark cycle. The mice were handled for 2–3 days to adapt to experimental conditions. All experimental procedures were approved by the local ethical committee and the Institutional Review Committee on Animal Care and Use (IACUC of AMMS-06-2017-003). 

The anesthetic effects of BZDs were measured using a mouse LORR model. The mice were tested individually in a clear plastic cage (40 × 20 × 20 cm, l × w × h). After BZD injection, the mice were gently placed in the supine position and the righting reflex was assessed every 1 min until the occurrence of LORR, which manifested as the failure to right themselves within 60 s. The anesthetized mice were left undisturbed until they spontaneously turned over themselves to prone position. Absolute recovery was defined as the mice being able to right themselves twice or more within 60 s. The time between BZD injection and occurrence of LORR was recorded as latency to LORR, and LORR duration was measured as the time from the occurrence of LORR to recovery. The LORR was considered absent if the mice were able to right themselves during the 120 min observation period after BZD injection.

### 4.5. Statistical Analysis

Electrophysiological data were analyzed with Clampfit 10.3 (Axon Instruments, San Jose, USA), Origin 8.0 (OriginLab Corporation, Northampton, MA, USA) and GraphPad Prism 5.0 (GraphPad Software Inc, La Jolla, CA, USA). Responses were normalized to the maximal response elicited by GABA. All data were presented as mean ± SEM, comparisons between groups were analyzed using one-way analysis of variance (ANOVA) with Dunnett’s post hoc test or unpaired *t*-test, and significant differences were considered if *p* < 0.05.

## Data Availability

The data from this study are available from the corresponding authors upon reasonable request.

## References

[1] Sieghart W., Sperk G. (2002). Subunit composition, distribution and function of GABA(A) receptor subtypes. Curr. Top Med. Chem..

[2] Baur R., Minier F., Sigel E. (2006). A GABA(A) receptor of defined subunit composition and positioning: Concatenation of five subunits. FEBS Lett..

[3] Farrant M., Nusser Z. (2005). Variations on an inhibitory theme: Phasic and tonic activation of GABA(A) receptors. Nat. Rev. Neurosci..

[4] Jacob T.C., Moss S.J., Jurd R. (2008). GABA(A) receptor trafficking and its role in the dynamic modulation of neuronal inhibition. Nat. Rev. Neurosci..

[5] Olsen R.W. (2018). GABA(A) receptor: Positive and negative allosteric modulators. Neuropharmacology.

[6] Sigel E., Ernst M. (2018). The Benzodiazepine Binding Sites of GABA(A) Receptors. Trends Pharmacol. Sci..

[7] Che Has A.T., Absalom N., van Nieuwenhuijzen P.S., Clarkson A.N., Ahring P.K., Chebib M. (2016). Zolpidem is a potent stoichiometry-selective modulator of alpha1beta3 GABAA receptors: Evidence of a novel benzodiazepine site in the alpha1-alpha1 interface. Sci. Rep..

[8] Rudolph U., Knoflach F. (2011). Beyond classical benzodiazepines: Novel therapeutic potential of GABAA receptor subtypes. Nat. Rev. Drug Discov..

[9] Masiulis S., Desai R., Uchański T., Martin I.S., Laverty D., Karia D., Aricescu A.R. (2019). GABA(A) receptor signalling mechanisms revealed by structural pharmacology. Nature.

[10] Middendorp S., Maldifassi M.C., Baur R., Sigel E. (2019). Positive modulation of synaptic and extrasynaptic GABAA receptors by an antagonist of the high affinity benzodiazepine binding site. Neuropharmacology.

[11] Kim J.J., Gharpure A., Teng J., Zhuang Y., Howard R.J., Zhu S., Noviello C.M., Walsh R.M., Lindahl E., Hibbs R.E. (2020). Shared structural mechanisms of general anaesthetics and benzodiazepines. Nature.

[12] Drexler B., Zinser S., Hentschke H., Antkowiak B. (2010). Diazepam Decreases Action Potential Firing of Neocortical Neurons via Two Distinct Mechanisms. Anesth. Analg..

[13] Zhu S., Noviello C.M., Teng J., Walsh R.M., Kim J.J., Hibbs R.E. (2018). Structure of a human synaptic GABAA receptor. Nature.

[14] Chua H.C., Chebib M. (2017). GABA(A) receptors and the diversity in their structure and pharmacology. Adv. Pharmacol..

[15] Walters R.J., Hadley S.H., Morris K.D., Amin J. (2000). Benzodiazepines act on GABAA receptors via two distinct and separable mechanisms. Nat. Neurosci..

[16] Sieghart W. (2014). Allosteric modulation of GABAA receptors via multiple drug-binding sites. Adv. Pharmacol..

[17] Baur R., Tan K.R., Lüscher B.P., Gonthier A., Goeldner M., Sigel E. (2008). Covalent modification of GABAA receptor isoforms by a diazepam analogue provides evidence for a novel benzodiaz-epine binding site that prevents modulation by these drugs. J. Neurochem..

[18] Wongsamitkul N., Maldifassi M.C., Simeone X., Baur R., Ernst M., Sigel E. (2017). α subunits in GABAA receptors are dispensable for GABA and diazepam action. Sci. Rep..

[19] Lian J.J., Cao Y.Q., Li Y.L., Yu G., Su R.B. (2020). Flumazenil-Insensitive Benzodiazepine Effects in Recombinant αβ and Neuronal GABA(A) Receptors. Brain Sci..

[20] Cao Y., Yan H., Yu G., Su R. (2019). Flumazenil-insensitive benzodiazepine binding sites in GABAA receptors contribute to benzodiazepine-induced immo-bility in zebrafish larvae. Life Sci..

[21] Fernandez S.P., Karim N., Mewett K.N., Chebib M., Johnston G.A., Hanrahan J.R. (2012). Flavan-3-ol esters: New agents for exploring modulatory sites on GABA(A) receptors. Br. J. Pharmacol..

[22] Gunja N. (2013). The Clinical and Forensic Toxicology of Z-drugs. J. Med. Toxicol..

[23] Baird E.S., Hailey D.M. (1972). Delayed recovery from a sedative: Correlation of the plasma levels of diazepam with clinical effects after oral and intravenous administration. Br. J. Anaesth..

[24] Klockowski P.M., Levy G. (1988). Kinetics of drug action in disease states. XXIV. Pharmacodynamics of diazepam and its active me-tabolites in rats. J. Pharmacol. Exp. Ther..

[25] Ramerstorfer J., Furtmüller R., Sarto-Jackson I., Varagic Z., Sieghart W., Ernst M. (2011). The GABAA receptor α+β- interface: A novel target for subtype selective drugs. J. Neurosci..

[26] McGrath M., Hoyt H., Pence A., Forman S.A., Raines D.E. (2021). Selective actions of benzodiazepines at the transmembrane anaesthetic binding sites of the GABAA receptor: In vitro and in vivo studies. Br. J. Pharmacol..

[27] Akk G., Steinbach J.H. (2011). Structural studies of the actions of anesthetic drugs on the γ-aminobutyric acid type A receptor. Anesthesiology.

[28] McGrath M., Hoyt H., Pence A., Jayakar S.S., Zhou X., Forman S.A., Raines D.E. (2020). Competitive Antagonism of Etomidate Action by Diazepam: In Vitro GABAA Receptor and In Vivo Zebrafish Studies. Anesthesiology.

[29] Bonetti E.P., Pieri L., Cumin R., Schaffner R., Pieri M., Gamzu E.R., Müller R.K., Haefely W. (1982). Benzodiazepine antagonist Ro 15-1788: Neurological and behavioral effects. Psychopharmacology.

[30] Çelik T., Deniz G., Uzbay I.T., Palaoğlu Ö., Ayhan I.H. (1999). The effects of flumazenil on two way active avoidance and locomotor activity in diazepam-treated rats. Eur. Neuropsychopharmacol..

[31] Auta J., Costa E., Davis J., Guidotti A. (2005). Imidazenil: An antagonist of the sedative but not the anticonvulsant action of diazepam. Neuropharmacology.

[32] Maldifassi M.C., Baur R., Pierce D., Nourmahnad A., Forman S.A., Sigel E. (2016). Novel positive allosteric modulators of GABAA receptors with anesthetic activity. Sci. Rep..

[33] Zhuo R.-G., Peng P., Zheng J.-Q., Zhang Y.-L., Wen L., Wei X.-L., Ma X.-Y. (2017). The glycine hinge of transmembrane segment 2 modulates the subcellular localization and gating properties in TREK channels. Biochem. Biophys. Res. Commun..

